# Impact of Extreme Weather on Healthcare Utilization by People with HIV in Metropolitan Miami

**DOI:** 10.3390/ijerph18052442

**Published:** 2021-03-02

**Authors:** Daniel Samano, Shubhayu Saha, Taylor Corbin Kot, JoNell E. Potter, Lunthita M. Duthely

**Affiliations:** 1 Miller School of Medicine, Department of Neurological Surgery, University of Miami, Miami, FL 33136, USA; 2 Miller School of Medicine, Department of Obstetrics and Gynecology, University of Miami, Miami, FL 33136, USA; jpotter2@med.miami.edu (J.E.P.); lduthely@med.miami.edu (L.M.D.); 3 Miller School of Medicine, Department of Public Health Sciences, University of Miami, Miami, FL 33136, USA; tkot@umc.edu; 4 Rollins School of Public Health, Emory University, Atlanta, GA 30322, USA; ssaha4@emory.edu; 5 School of Medicine, University of Mississippi Medical Center, Jackson, MS 39216, USA

**Keywords:** extreme weather events, climate and health, climate change, healthcare access, HIV

## Abstract

Extreme weather events (EWE) are expected to increase as climate change intensifies, leaving coastal regions exposed to higher risks. South Florida has the highest HIV infection rate in the United States, and disruptions in clinic utilization due to extreme weather conditions could affect adherence to treatment and increase community transmission. The objective of this study was to identify the association between EWE and HIV-clinic attendance rates at a large academic medical system serving the Miami-Dade communities. The following methods were utilized: (1) Extreme heat index (EHI) and extreme precipitation (EP) were identified using daily observations from 1990–2019 that were collected at the Miami International Airport weather station located 3.6 miles from the studied HIV clinics. Data on hurricanes, coastal storms and flooding were collected from the National Oceanic and Atmospheric Administration Storms Database (NOAA) for Miami-Dade County. (2) An all-HIV clinic registry identified scheduled daily visits during the study period (hurricane seasons from 2017–2019). (3) Daily weather data were linked to the all-HIV clinic registry, where patients’ ‘no-show’ status was the variable of interest. (4) A time-stratified, case crossover model was used to estimate the relative risk of no-show on days with a high heat index, precipitation, and/or an extreme natural event. A total of 26,444 scheduled visits were analyzed during the 383-day study period. A steady increase in the relative risk of ‘no-show’ was observed in successive categories, with a 14% increase observed on days when the heat index was extreme compared to days with a relatively low EHI, 13% on days with EP compared to days with no EP, and 10% higher on days with a reported extreme weather event compared to days without such incident. This study represents a novel approach to improving local understanding of the impacts of EWE on the HIV-population’s utilization of healthcare, particularly when the frequency and intensity of EWE is expected to increase and disproportionately affect vulnerable populations. More studies are needed to understand the impact of EWE on routine outpatient settings.

## 1. Background

Science predicts an increase of 1.5 °C will lead to devastating extreme weather events (EWE), such as heavy rainfall or a high heat index [1]. Globally, EWEs are becoming more frequent and more intense [2], with coastal areas like Miami, Florida being particularly vulnerable [3]. The State of Florida (the warmest state in the United States (U.S.)) [4] is surrounded by water and had a documented annual mean temperature increase from 69.8 °F in 1895 to 71.2 °F in 2015—an increase of 1.4 °F over 120 years. However, individual regions within a state may experience changes that differ from the mean. Miami has experienced a steady increase in its annual average temperature, from 75.1 °F in 1950 to 77.7 °F in 2017—a 2.6 °F increase over 67 years—and temperatures 70 years from now are expected to rise above 95 °F 45 to 90 days per year [5]. From 1950–2017, the daily precipitation (DP) increased from 44.1 inches to 53.8 inches for Miami, Florida [6]. Several studies have found associations between emergency department visits or emergency preparedness and weather events; heat waves, for example, have been associated with higher numbers of emergency department visits in the United States [7]. Little is known, however, regarding healthcare utilization during EWEs in non-emergency settings. It is important to understand EWE within the context of healthcare utilization in outpatient settings, where appointments can be re-scheduled or cancelled with more flexibility than an emergency room visit and the medical needs of the patients can be addressed.

All persons within a community are vulnerable to EWE, though the level of vulnerability is greater among those already vulnerable with regards to co-occurring health conditions. Another consideration is the disruption of access to treatment for those with infectious diseases. Metropolitan Miami (Miami) has the highest national average of HIV transmission in the United States (US), and adherence to both medical appointments and treatment is crucial to improve health outcomes in reducing the risk of transmission in the community [8].

This study builds on previous research [9,10,11], indicating a trend toward lower clinic attendance on high heat days and high precipitation (i.e., rainy) days. The current study sought to measure the association between different types of extreme weather events and HIV clinic utilization patterns in Miami by answering the following question: to what extent does EWE impact the healthcare utilization habits of people living with HIV (PLWH) in Miami, Florida? We hypothesized a higher significant difference in clinic non-attendance on non-extreme vs. extreme weather days.

## 2. Methods

**Figure 1:**
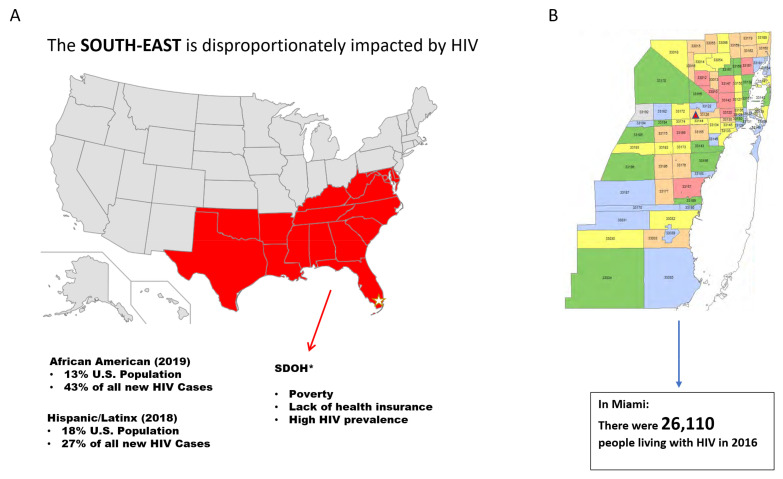
The importance of studying people living with HIV. (**A**). United States map highlighting southeastern states (red). Yellow Star indicates Miami Dade County, in the State of Florida. SDOH* Social Determinants of Health. (**B**). Miami-Dade County by zip codes, the red triangle indicates the location of the Miami International Airport.

In order to derive locally relevant weather information, we first calculated the distribution of extreme precipitation (EP) and extreme heat index (EHI) [12] using data (1999–2019) from a National Weather Service station located at the Miami International Airport (Figure 1B). This weather station is located 3.6 miles from the institution where the HIV clinics are located. Review of the integrated surface data report [13], and the Local Climatological Dataset Documentation [14] was necessary to understand the robust National Oceanic and Atmospheric Administration (NOAA) database variables and their definitions. We used hourly observations of temperature (T) and relative humidity (RH) information to calculate the heat index using the Rothfusz multiple regression formula, as shown in Appendix A [15]. Each day was classified into one of five EHI categories (<90, 90–95, 95–100, 100–105, >105 °F). For comparison, the 90th percentile of the daily heat index distribution over the 30-year period was at 100.83 °F. We added up the 24 h precipitation measurements to calculate daily precipitation. Each day was classified into one of four EP categories (0, 0–1, 1–2, >2 inches). For comparison, the 90th percentile of the daily precipitation distribution over the 30-year period was at 2.01 inches. We used absolute values for heat index and precipitation to create these categories, as it would be easier to communicate this to healthcare professionals and general public. For specific extreme events, we used the NOAA storms database to identify dates where there was a recorded ‘flood’, ‘hurricane’, or ‘tropical’ storm that affected the Miami-Dade County during the study period [16].

In Miami-Dade County (MDC), the care of approximately 10% of the population of PLWH (approximately 28,000 in 2018) [17] is concentrated in one academic medical system. The large non-profit organization and county-owned academic medical system serves north, central, and south MDC, with a patient service area that covers 49 zip codes and includes over 1,963,942 residents. MDC represents 13% of Florida residents, and is the 8th largest county in the US (Figure 1B). It is one of the few counties in the US that is “minority–majority”, in that a minority group comprises the majority of the population. As identified by its main payers (Medicaid (44.9%) and Medicare (23.1%)), our institution provides health care through specialized primary care clinics and OB/GYN HIV clinics to the most vulnerable populations in MDC [18].

Our institution’s HIV services provide integral medical care to all adult PLWH through six clinics. An all-HIV clinic registry database was created from a large county hospital in South Florida, in collaboration with the department data manager. Clinics’ scheduled data were de-identified and organized into the following categories: attended, not attended, re-scheduled, lost, canceled, and no-show. ‘No-show’ was the variable of interest, as these patients failed to notify the HIV clinic about their appointment re-schedule or cancellation. This database runs during hurricane season (from May to October) from the years 2017–2019 during clinic operating hours from 8:00 am to 5:00 pm, excluding weekends, holidays, and any other non-operating clinic days (i.e., Hurricane Irma in September 2017).

We used a time-stratified case crossover approach to assess how clinic visits were impacted on days with adverse weather (high heat index, high precipitation, and specific extreme events). We controlled for the day of the week in a given month and compared the rate of no-show on days with adverse conditions to days with no adverse conditions. We used a Conditional Poisson regression model [19] to estimate the relative risk of no-show on days with high heat index, precipitation, and/or an specific extreme natural event, separately. The reference category used in the analysis referred to the heat index on days <90 °F, with no precipitation and no extreme natural event. Analyses were performed in SAS, R, and Microsoft Excel.

## 3. Results

Based on 30 years of data, the EWE values were as follows: the EHI values corresponding to any value above the 90th percentile was 100.83 °F (Appendix A–Figure A1A,B); the Extreme Precipitation amounts corresponding to any value above the 90th percentile was 2.01 inches per day (Appendix A–Figure A1C,D).

**Figure 2:**
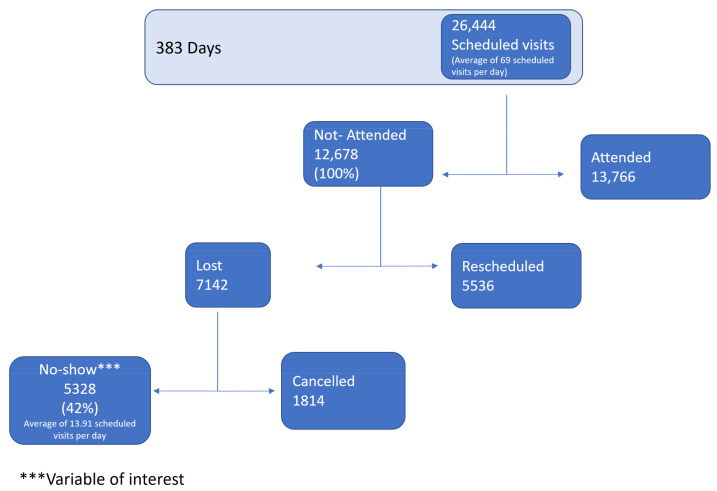
All HIV clinic registry.

There were 26,444 scheduled visits by the 383th day of the study period, equivalent to 26,444 scheduled visits and an average of 69 scheduled visits per day. From the 12,679 (100%) that had not attended their appointment, the no-show subset was 5328 (42%) (Figure 2).

**Figure 3:**
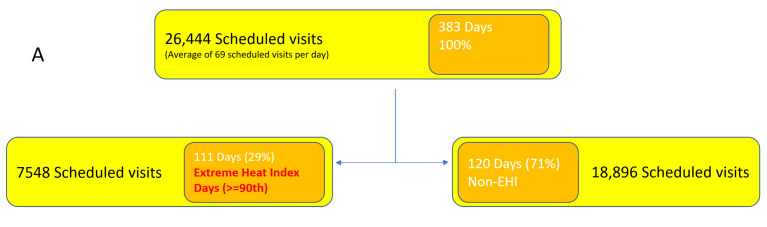

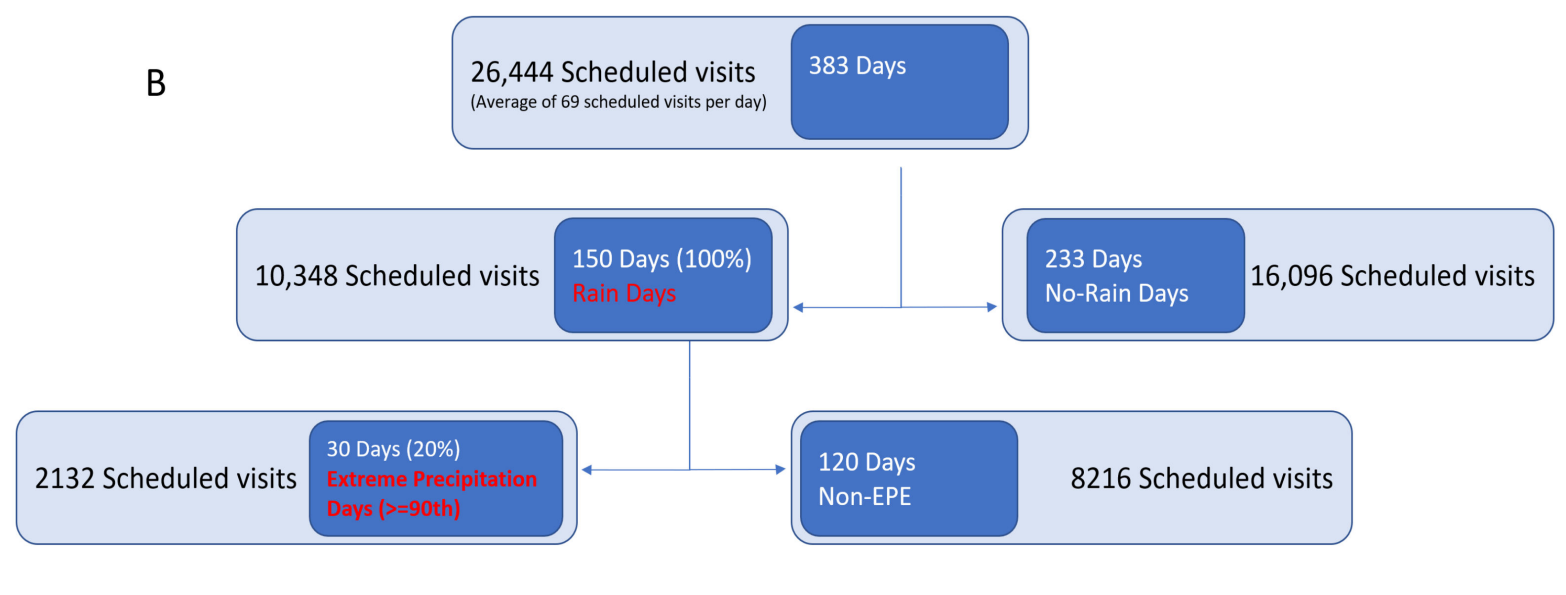
Weather and extreme weather events during study period. (**A**): Number of days with and without an extreme heat index in the study period. (**B**): Number of days with and without extreme precipitation in the study period.

There were 111 (29%) days with an extreme heat index in the study period, equivalent to 7548 scheduled visits to an HIV clinic (Figure 3A), and 150 (39%) days with rain in the study period, equivalent to 10,348 scheduled visits. The sum of the number of extreme precipitation days was equivalent to 2132 scheduled visits to an HIV clinic (Figure 3B). The distribution during the study period of daily heat index and precipitation is shown in Table 1 and Table 2, respectively. For a majority of the days, the heat index was in the 95–105 °F range, and there was either no rain or less than an inch of rain. An extreme event happened for about 10% of the days in the study period.

**Figure 4:**
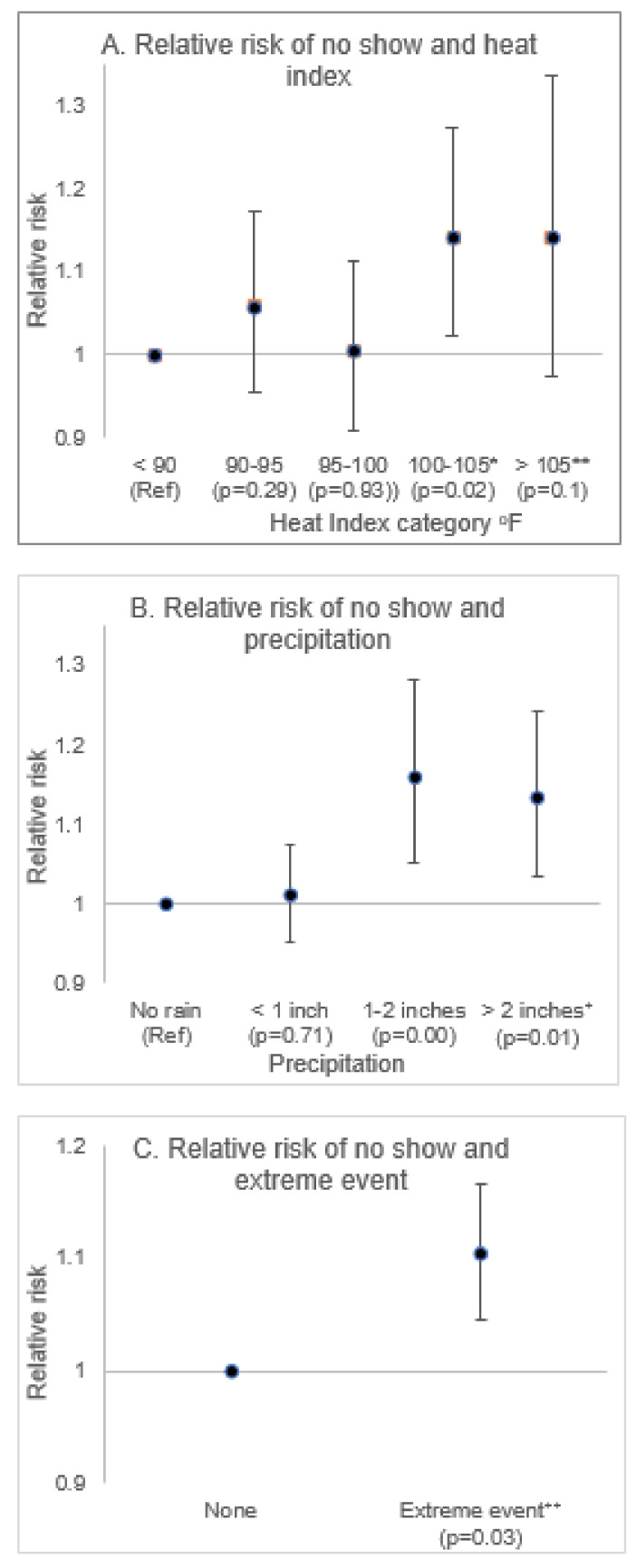
The relative risk of ‘no-show’ visits associated with extreme weather events (EWE). (**A**). Relative risk of ‘no show’ and heat index by categories. * Category: 100–105 °F; in this study, the extreme heat index (EHI) for Miami is a value of ≥100.83 °F (90th percentile), and a value of 102.75 °F (95th percentile) is also within this category. ** EHI ≥104.58 °F (97th percentile) is nearly starting at this category. (**B**). The relative risk of ‘no show’ and precipitation by categories. ⁺ Category: >2 inches; in this study, extreme precipitation (EP) in Miami is considered to have a value of ≥2.01 inches (90th percentile). (**C**). The relative risk of no show and extreme event. ⁺⁺ Extreme event referring specifically for ‘flood’, ‘hurricane’, and ‘tropical storm’ events (National Oceanic and Atmospheric Administration (NOAA) storms database).

The results from the regression model with ‘no-show’ as the dependent variable are presented in Figure 4. Compared to days when the heat index was below 90 °F, a steady increase in the relative risk of ‘no-show’ was observed at successive heat index categories, with a 14% increase observed on days when the heat index was above 100 °F. Similarly, compared to days with no precipitation, the relative risk of ‘no-show’ was higher on days when precipitation was above an inch. Specifically, the relative risk increased by 16% on days with 1–2 inches of precipitation and 13% on days with greater than 2 inches of precipitation. The risk of ‘no-show’ visits was 10% higher on days with a reported extreme natural event compared to days when no such incident was reported.

## 4. Discussion

To the authors’ knowledge, this is the first study ever published demonstrating an association between extreme weather events (specifically EHI and EP) with lower outpatient healthcare visits. Little is known about what climate change may mean for access to healthcare in general. This study serves as a starting point to measure the expected subtle but progressive impact of EWE on healthcare utilization, particularly in outpatient clinic settings. The study focus was persons with HIV (a chronic and infectious condition requiring regular care and treatment) who were followed in Metropolitan Miami—the epicenter of new HIV infections in the US. Among the vulnerable groups of persons in the setting of climate change and its consequences are those with immunodeficiency, including those with HIV infection [20]. In addition to the burden faced by an individual living with an HIV diagnosis is the fact that many subgroups of PLWH (e.g., racial/ethnic, sexual, and gender minorities, persons living in poverty, and persons living in rural regions) face an added burden of the social determinants of their health outcomes, which include racial/ethnic background, income level, and where they live [8]. The United Nations seeks to end the HIV/AIDS epidemic by 2030, setting the “90–90–90” targets of 90% of persons with HIV being diagnosed, 90% of those diagnosed being in treatment, and 90% of those treated being virologically suppressed [21]. The HIV/AIDS epidemic is a global public health crisis that disproportionately affects different population sub-groups and different regions. For Miami-Dade County, the HIV/AIDS treatment cascade is as follows: only 64% of diagnosed PLWH are in regular care, and only 60% of those in care were virologically suppressed—viral suppression prevents disease progression and prevents the transmission of the HIV virus in the community [17]. Understanding the influence of EWE on the healthcare for PLWH may increasingly become an important factor towards better control of the HIV epidemic.

PLWH are a particularly vulnerable group in this context. The primary and secondary prevention of HIV represents one of the biggest challenges in this century worldwide [22]. In the Southeastern US, the HIV epidemic prevalence and incidence is alarming [23]. African American (AA) and Hispanic/Latinx individuals represent nearly 70% of all HIV cases [8], characterized by lack of health insurance and living in poverty [8]. MDC houses one of the three cities with the highest HIV diagnosis rates [8]; in 2016, there were 1246 new diagnoses of HIV, with a vast majority of these being Hispanic and AA individuals [24]. MDC has a poverty rate of 18.3%, nearly 30% higher than the state and national averages [25]. Hurricane season is of importance to the MDC community, as several preparedness efforts take place during these months. The fact that this study found nearly 30% of days to have extreme heat and 20% of days to have extreme precipitation during three consecutive years’ hurricane seasons (Figure 3) may represent another burden to the population of study.

Patients not adherent to medical appointments are more likely to not adhere to their medications, thereby increasing the risk of transmission of HIV to sexual partners (men and women) and unborn babies (reproductive-aged women). From a clinic–policy perspective, for example, a systematic process evaluating weather and predicted weather or clinic operations such as extended hours or weekend dates could be modified to minimize clinic attendance disruption while mitigating potential exposure to an EWE. The use of technology could alleviate the burden of health systems; for example, through the implementation of tele-medicine, facilitating a home care discussion. These options, however, would need careful consideration to balance the availability of resources.

The recent US National Climate Assessment highlights the increase in climate risk that communities in the Southeastern regions face from extreme weather, including extreme heat and extreme flooding. These climate risks pose new challenges to patients seeking routine healthcare, as well as for professionals providing these services [26]. As a way to mitigate the effects of climate risk on healthcare, it has been suggested to utilize locally-specific health evidence to inform heat alert criteria [27]. Healthcare providers, institutional policymakers, and local and regional policymakers can prepare for an EWE day using the risk information we provide from our analysis in combination with forecasts of extreme weather. For example, the NOAA heat index chart [28] can be used a point of reference by HIV clinics to inform patients and staffing schedules by combining the risk information with a forecast of extreme heat. Our findings do not negate NOAA’s heat index chart; rather, our results amplify the need for a localized definition of “extreme” which is grounded within the context of regional data. The results of the analysis have implications regarding how information on adverse weather can be effectively utilized in the planning and delivery of routine healthcare. Our study demonstrated changing weather patterns in Miami, located in the southeast region of the State of Florida—the warmest state in the US (4)—which has become progressively warmer and “rainier” since 1950 (6). This pattern is expected to progressively worsen annually (5). Our study quantified extreme values during Miami’s hurricane season from 2017–2019, with nearly 30% of the days displaying extreme heat and 20% of the days displaying extreme precipitation, demonstrating how healthcare utilization was impacted by EWE.

Health risk assessments like these will help identify a range of adverse weather conditions, which could inform the calibration of health-weather alerts. For example, we observed a statistically significant decline in clinic visits on days below the threshold of excessive heat warnings issued by the National Weather Service for the Miami region. We need to factor in the frequency and intensity of EWE, which could make the public health crisis worse. Analyses like ours can help start a dialogue among critical stakeholders to re-plan their HIV population’s adherence to treatment, reassess current efforts on extreme weather days, and train their providers about the impact of climate change on PLWH. This study strength resides in the link between climate and health with a methodology to derive weather extremes and test their associations with healthcare utilization patterns by geographic location. The implemented methods could be replicated in different geographic locations and could be extended to clinical service areas, other than HIV clinics at one institution.

As an observational, descriptive, ecological study, this project has the strengths and weaknesses inherent in the design, including the ecological fallacy—the observed association between EWE and healthcare utilization by PLWH does not necessarily represent the association that exists at the individual level of someone living with HIV in Miami. There are several limitations that must be noted; first, because of limitations of the health data available, we were not able to control for important patient-level characteristics that can affect clinic visits. While the results of the impact of extreme weather on clinic visits are novel, they only partially explain the observed fluctuations in clinic visits. No causality can be established between the EWE and healthcare utilization. Precipitation data focuses on rainfall measurements during working hours, and does not include precipitation outside of working hours or daily precipitation data. The latter represents a limitation to this study, since flooding was not being considered.

## 5. Conclusions

As the effects of climate change intensify, there is an increased need to understand how extreme weather patterns affect healthcare utilization by geographic location, given the potential for the disruption of medical treatment for individuals with the greatest need—patients reliant on treatment for their health conditions and persons with infectious diseases. This is the first study documenting a weather methodology in relation to healthcare utilization patterns overall, which was detailed within PLWH—a group dependent on treatment for their own health outcomes and their ability to avoid the further spread of HIV to others.

From this study, we were able to derive a local definition for both an EHI and EDP, and a methodology to be able to replicate this approach at any location’s EWE with any patient clinic registry, given that weather data are publicly available. Our data have demonstrated an association between EWEs (specifically heat index and precipitation) with the healthcare utilization of PLWH. Even without considering weather events, these data highlight the challenges in terms of adherence to care in the study population. Large clinics with irregular attendance patterns may want to assess clinic attendance and explore the factors that may be driving increasing rates of patients not attending clinics, with the goal of improving healthcare utilization and patient health outcomes.

Our findings demonstrate a significant inverse association between EHI and EP and clinic attendance. More studies are needed to identify the subtle but progressive influence of climate on health. Solutions are needed to provide care for PLWH and determine how to mitigate these potential effects through public health capacity building and collective resiliency.

## Figures and Tables

**Table 1:** Distribution of the daily heat index.

	Frequency	Percent	Relative Risk	95% C.I.		Pr(>|z|)
Category 1: <90 °F	38	9.92	Ref			
Category 2: 90–95 °F	70	18.28	1.06	0.95	1.17	0.29
Category 3: 95–100 °F	128	33.42	1.00	0.91	1.11	0.93
Category 4: 100–105 °F	128	33.42	1.14	1.02	1.27	0.02
Category 5: >105 °F	19	4.96	1.14	0.97	1.34	0.10

**Table 2:** Distribution of daily precipitation.

Precipitation	Frequency	Percent	Relative Risk	95% C.I.		Pr(>|z|)
Category 1: None	233	60.84	1.01	0.95	1.07	0.71
Category 2: <1 inch	101	26.37	1.16	1.05	1.28	0.00
Category 3: 1–2 inches	19	4.96	1.13	1.04	1.24	0.01
Category 4: >2 inches	30	7.83	1.01	0.95	1.07	0.71

**Figure A1:**
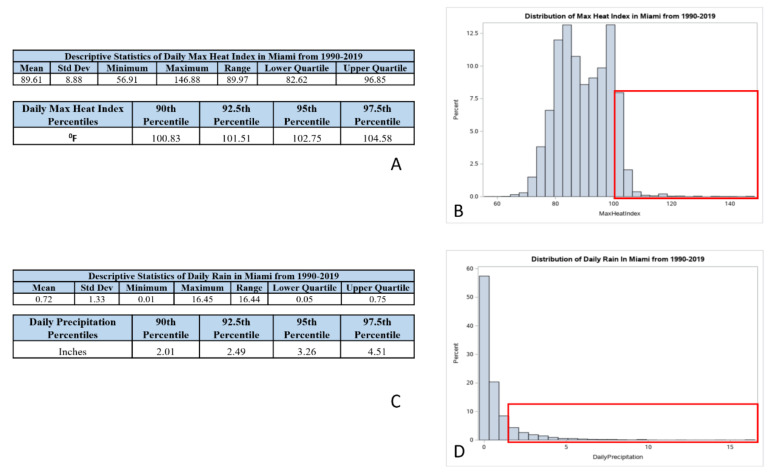
1990–2019 Retrospective analysis of NOAA weather data collected every hour from January 1990 to October 2019 at Miami International Airport’s zip code, located 3.5 miles from the institution. (**A**): SAS software analysis from the maximum daily Heat Index Calculation*. Top= Descriptive statistics: mean, median, minimum, maximum, standard deviation, and lower and upper quartiles. Bottom= Percentiles (1): 90th, 92.5th, 95th, and 97.5th. (**B**): A maximum heat index normality curve with a bell-shaped bimodal presentation. Red boxes indicate all values that are considered extreme at Miami International Airport weather station’s (MIAs) zip code 33142, located 3.6 miles from the institution location. (**C**): SAS Analysis from daily hourly-precipitation. Top= Descriptive statistics: mean, median, minimum, maximum, standard deviation, lower and upper quartiles. Bottom= Percentiles (1): 90th, 92.5th, 95th, and 97.5th. (**D**): Daily rain normality curve. Distribution bell shape is not presented, as this is a binary variable. Red boxes indicate all values that are considered extreme at Miami International Airport weather station’s (MIAs) zip code (33142), located 3.6 miles from the institution location. * Heat Index (HI) was calculated using the data points of temperature (T) and relative humidity (RH) by the computation of Rothfusz multiple regression formula (HI = −42.379 + 2.04901523 × T + 10.14333127 × RH − 0.22475541 × T × RH − 0.00683783 × T × T − 0.05481717 × RH × RH + 0.00122874 × T × T × RH + 0.00085282 × T × RH × RH − 0.00000199 × T × T × RH × RH. The heat index formula was adjusted as suggested by NOAA: if “RH is less than 13% and the temperature is between 80 and 112 degrees F”, then ADJUSTMENT = [(13-RH)/4] × SQRT{[17-ABS(T-95.)]/17} where ABS is absolute value and SQRT is square rot functions. However, if “RH is greater than 85% and the temperature is between 80 and 87 degrees F”, then ADJUSTMENT = [(RH-85)/10] × [(87-T)/5]).

## Data Availability

Publicly available datasets were analyzed in this study. This data can be found at https://www.ncdc.noaa.gov/cdo-web/ (accessed on 1 January 2021). Clinical registry data presented in this study should be directed to the primary author’s Privacy Office (http://privacy.med.miami.edu/contact (accessed on 1 January 2021)).
